# MULTIPLE CHRONIC CONDITIONS AND RISK OF DEMENTIA AND COGNITIVE IMPAIRMENT

**DOI:** 10.1093/geroni/igz038.1156

**Published:** 2019-11-08

**Authors:** Yura Lee, Youngjoo Cho, Hyunkyoung Oh

**Affiliations:** 1 University of Wisconsin - Milwaukee, Milwaukee, Wisconsin, United States; 2 University of Wisconsin-Milwaukee College of Nursing, Milwaukee, Wisconsin, United States

## Abstract

This study explores the relationship between the presence of multiple chronic condition and risk of dementia and cognitive impairment with no dementia (CIND) among older Americans. The study sample included 617 participants aged 70 years and older from the Aging, Demographics, and Memory Study (ADAMS). An expert consensus panel of the ADAMS data provided each participant a cognitive diagnosis into 1) no cognitive impairment, 2) CIND, or 3) dementia. The presence of multiple chronic condition was defined as having three or more chronic conditions in this study (e.g., heart attack, stroke, respiratory problems, cancer, hypertension, diabetes). Functional limitation, depression, cognitive activity engagement, apolipoprotein E (ApoE), and sociodemographic characteristics were included as covariates. A multinomial logistic regression analysis showed that individuals who have multiple chronic conditions have increased odds of being diagnosed with CIND versus no cognitive impairment controlling for other covariates. However, multiple chronic condition was not associated with increased risk of being diagnosed with dementia versus no cognitive impairment. Conclusion: The finding suggests that the presence of multiple chronic conditions may be a risk factor for cognitive impairment in later life. However, further investigation using a longitudinal design is needed to better understand the relationship between cognition and multiple chronic conditions.

